# An Atypical Presentation of Pediatric Acute Neuropsychiatric Syndrome Responding to Plasmapheresis Treatment

**DOI:** 10.1155/2018/8189067

**Published:** 2018-06-28

**Authors:** Drew H. Barzman, Hannah Jackson, Umesh Singh, Marcus Griffey, Michael Sorter, Jonathan A. Bernstein

**Affiliations:** ^1^University of Cincinnati College of Medicine, Department of Psychiatry and Behavioral Neuroscience, CARE/Crawley Building, Suite E-870 3230 Eden Avenue Cincinnati, OH 45267, USA; ^2^Cincinnati Children's Hospital Medical Center, 3333 Burnet Avenue, Cincinnati, OH 45229-3026, USA; ^3^University of Dayton, 300 College Park, Dayton, OH 45469, USA; ^4^University of Cincinnati College of Medicine, Division of Immunology Allergy Section, 231 Albert Sabin Way, Cincinnati, OH 45267, USA; ^5^University of Cincinnati College of Medicine, Department of Pediatrics, 231 Albert Sabin Way, Cincinnati, OH 45267, USA

## Abstract

Here we report a case of a 15-year-old female who had originally been diagnosed and treated unsuccessfully for schizophrenia, psychosis, severe anxiety, and depression. More in-depth history revealed an abrupt onset of her symptoms with remote acute infections and many exhibited characteristics of obsessive compulsive disorder with rituals. Work-up for underlying infectious, immunodeficiency, and autoimmune causes was unrevealing except for very high levels of anti-neuronal antibodies which have been linked to Pediatric Acute-onset Neuropsychiatric Syndrome (PANS). Treatment options were discussed with the family and it was decided to use a course of plasmapheresis based on previous studies demonstrating efficacy and its safety profile. After course of therapy, there was a dramatic resolution of her psychosis, OCD traits, and anxiety. She was able to stop all of her antipsychotic and anxiety medications and resume many of her previous normal daily activities. The effect of this treatment has been sustained to the present time. This case emphasizes the importance of exploring nontraditional treatments for severe, treatment-resistant mental illness which requires a multidisciplinary approach. Further research is warranted in larger populations to investigate pathomechanisms and treatment of PANs/PANDAs.

## 1. Case Presentation

Patient A presented as a mostly healthy 15-year-old Caucasian female with some developmental disabilities and ADHD, characterized by poor attention span, poor attention to details, poor organization, forgetfulness, excessive talking, impulsivity, and distractibility since age seven. Her father reported two severe brain injuries around the age of five. Over the course of one year at age 15, she required four inpatient psychiatric hospitalizations and numerous outpatient and medication management appointments due to an acute onset of “seizure-like” spells, psychotic thinking, and seemingly schizophrenic symptoms, manifesting as auditory hallucinations (AH) and catatonic movements. The differential diagnosis included schizophrenia, severe Tourette syndrome, Major Depressive Disorder, Obsessive Compulsive Disorder, and Posttraumatic Stress Disorder.

## 2. Clinical Course

Over time, Patient A had several strange physical symptoms including dysphonia, mouth twitches, echolalia, frequent pacing, frequent cussing, holding her breath, repeatedly asking the same questions, crying and laughing for no reason, staring, outstretching of her arms for 30 minutes, stumbling, worsening dysgraphia, unable to solve math problem, and worsening reading skills.

Initially, the change in her behavior was thought to be a neurologic issue due to the “seizure-like” spells, characterized by uncontrollable mouth twitching, eye rolling, and staring into space. However, after an unrevealing neurology evaluation she was referred to psychiatry. Mood and anxiety disorders were also suspected due to fears of social situations, making mistakes, and trying new things in conjunction with irritability, muscle tension, insomnia, self-consciousness, stomachaches, and feelings of worthlessness resulting in self-blame.

After a few months of declining mental health, patient A began outpatient psychotherapy sessions, where she discussed issues with being bullied and social anxiety at school. During these sessions, patient A's professional clinical counselor (LPCC) consistently noted she was zoning out, mouthing words silently, seemingly in response to internal stimuli, and exhibiting unilateral catatonic right arm movements.

Due to the lack of outpatient success, patient A was admitted to a partial hospitalization program (PHP). There, she displayed symptoms of mouthing words and laughing as a response to internal stimuli, outbursts of cussing at friends not present, leaving food in mouth for hours before swallowing, and deterioration of handwriting. Due to the severity of symptoms, patient A was admitted to an inpatient psychiatry unit, where she was diagnosed with a psychotic disorder. Interestingly, she had experienced a Streptococcus infection one month prior to this first admission. While on the inpatient unit for eight days, risperidone 0.25 mg BID was started and sequentially increased to 0.5 mg QAM and 1.0 mg QPM, which caused enough improvement for patient A to return to the PHP. All neuroleptic trials for this patient lasted for about six to eight weeks.

The prevalence of her auditory hallucinations (AH's) increased in quantity and severity while in the PHP, so she was admitted a second time to inpatient psychiatry, where she began treatment for psychosis and schizophrenia. During this admission, she admitted that some of the voices in her head were her own and another voice was a male bully from school telling patient A to kill herself, raising questions as to whether these were actual AHs or flashbacks from past traumatic experiences.

Unfortunately, patient A continued to have difficulty with chewing and swallowing food, which led to gagging, choking, and emesis, as well as echolalia, restlessness, inappropriate smiling, and irregular arm movements. Her medication regimen was further altered to include benztropine 0.5 mg BID, ziprasidone 20 mg BID, and trazodone 25-50 mg at night for sleep. On this medication regimen, patient A showed improvement for the first few days before the AHs and other symptoms began to once again hinder her daily function.

After almost two weeks of crisis stabilization, patient A was discharged and sent back to the PHP, where thought blocking, flat affect, responding to internal stimuli, anxiety, and jerking movements persisted. She then began having self-harm and suicidal thoughts. Ziprasidone was increased to 40 mg QAM and 80 mg QPM with the goal of relieving the AHs while minimizing psychotic and seemingly schizophrenic symptoms. Propranolol 10 mg was introduced as needed for agitation. After little improvement, she was admitted to inpatient for a third time for a higher level of care.

During this hospitalization, ziprasidone was cross-tapered with aripriprazole with little benefit. Aripriprazole was then cross-tapered with lurasidone up to a dose of 40 mg QAM. Benztropine was increased to 0.75 mg BID and lorazepam to 0.25 mg BID and 0.5 mg QHS was also started. The as needed trazodone and propranolol were discontinued. With this regimen, AHs were only reported every other day, and activities of daily living (ADL) were able to be completed with less anxiety and prompting from staff, allowing her to be discharged from her third inpatient hospitalization.

Patient A continued to have episodes of acute psychosis with AHs each lasting between 30 and 150 minutes three to four times a week. She also had frequent emesis associated with taking lurasidone while at home. Her physicians suggested starting her on Clozaril, a medication for severe treatment-resistant schizophrenia, due to only partial effectiveness of lurasidone, but her parents declined due to required weekly blood draws for monitoring absolute neutrophil blood counts. After four failed antipsychotic medications, the psychiatry team chose to start olanzapine 5 mg TID in place of lurasidone. Benztropine 1 mg BID was continued and clonazepam 0.25 mg TID for longer-lasting anxiety relief was also implemented.

However, patient A required a fourth inpatient hospitalization due to anxiety attacks, suicidal ideation, and continuous “scary” thoughts. Schizophrenia and psychotic disorder continued to be the diagnoses, even after the four failed courses of antipsychotic drugs. She continued to have significant difficulty with her activities of daily living and anxiety, which caused a constant sad and fearful state. The medication regimen was altered to reintroduce Geodon, so now she was taking benztropine 1 mg BID, clonazepam 0.25 mg BID, clonazepam 0.5 mg QHS, olanzapine 5 mg QAM, olanzapine 7.5 mg QPM, ziprasidone 20 mg BID, and a daily multivitamin.

After ten days, patient A was discharged from inpatient psychiatry after demonstrating overall improvement even though she still had issues with muscle stiffness, reduction in speech, and anhedonia at home. After a short time of improvement, the AH's returned as did showering excessively and pacing for 3-8 hours a day. At this point, quetiapine 50 mg QAM and 100 mg QPM was added to the medication regimen.

During frequent phone conversations with patient A's mother and father over the next few months, it was noted that there was a family history of OCD with tics (mother) and Autism Spectrum Disorder (maternal uncle). Her mother reported that patient A's symptoms began to increase in occurrence and presented in a fashion consistent with OCD. Patient A exhibited varying tics such as repeatedly muttering the same word in an almost inaudible tone or screeching as loud as she can with her mouth open, excessive chewing on anything she could put into her mouth, turning light switches off and on, walking halfway up a flight of stairs, and then going back down in a ritualistic fashion. It became increasingly likely that patient A's yearlong issues were related to OCD and tics, rather than schizophrenia and psychosis. To further evaluate for OCD, she was assessed with the Yale Brown Obsessive Compulsive Scale, a validated rating scale, which provided evidence for a diagnosis of OCD with a moderate to severe range [1]. No other scales for tics or psychosis were completed. The results of this evaluation, along with her family history of OCD, led to the decision to treat her condition as OCD and anxiety. She was weaned off all antipsychotic medications and began taking clonazepam (0.25 mg TID) and fluoxetine (20 mg QAM). After the decrease in olanzapine, she exhibited much more energy, normal appetite, better sleep, and reduced tics. A neurologist subsequently evaluated patient A for unilateral arm movements which was felt to be only a temporary drug-induced extrapyramidal symptom. The neurologist also ruled out any neurologic disorders including autoimmune pediatric neuropsychiatric disorder associated with streptococcal infection (PANDAS) due to the lack of a verified streptococcus infection in her past medical history and normal ASO titers.

After a period of consistent improvement in her OCD symptoms on Prozac, she began to regress with recurrence of severe AHs, delusions, and poor functioning, which led to a 6-week inpatient hospital stay. During this admission, patient A presented with different symptoms such as stumbling or falling over, dysgraphia, and losing the ability to complete math problems and read without assistance. The psychiatrist ordered consultations by neurology, immunology, rheumatology, and an OCD expert due to suspicions of Pediatric Acute-onset Neuropsychiatric Syndrome (PANS). The consultants did not find any evidence for PANS and advised against treatment as laboratory testing for autoimmune encephalitis (i.e., anti-N-Methyl-D-Aspartate (NMDA) receptor antibodies), other autoimmune disorders, and two brain MRIs were all normal. However, the psychiatrist still believed that an atypical autoimmune mediated process could be the cause of her neuropsychiatric symptoms due to the atypical presentation and treatment resistance to medications.

After her discharge from the inpatient service, patient A was referred to an adult academic immunologist for a second opinion. A Cunningham panel was ordered, which measures anti-neuronal IgG antibodies (Cunningham panel) directed against dopamine 1 and 2 receptors, lysoganglioside-GM1, tubulin, and calcium/calmodulin-dependent protein kinase II (CaM KII). The results of this test revealed significant elevation of the first four antibodies and a borderline increase for CaM KII (Table 1) [2]. Based on the patient's history and laboratory testing, it was felt she fulfilled the criteria for a diagnosis of PANS [2]. Given her diagnosis of PANS and lack of success with other treatment approaches, the psychiatrist, immunologist, and parents determined that the potential benefits for treatment of PANS were warranted. After discussion of treatment options with the parents, it was decided that plasmapheresis to remove the anti-neuronal antibodies would be most appropriate treatment based on its safety and previous small studies demonstrating its efficacy [3, 4]. After one course of plasmapheresis was administered (seven treatments every other day), the patient had complete resolution of her psychotic, OCD, and anxiety symptoms. She was able to be weaned off olanzapine and resume many of her normal activities including tennis, within 2 weeks after plasmapheresis. This response has now been sustained for over six months consistent with previous studies [5]. Unfortunately, due to cost, it was not possible to check postplasmapheresis anti-neuronal antibody levels.

## 3. Discussion

Previous studies have reported an association between specific bacterial or viral infections, positive anti-neuronal antibody titers, and sudden onset neuropsychiatric dysfunction (e.g., OCD) in subgroups of children [2, 5, 6]. These clinical syndromes have been broadly classified as Childhood Acute Neuropsychiatric Symptoms (CANS) and subclassified as Pediatric Acute-onset Neuropsychiatric Syndrome (PANS) to encompass all infectious agents (19% of reported case including Borrelia. burgdorferi, Mycoplasma. pneumonia, and Herpes simplex) and more specifically PANDAS which is associated with streptococcal infection (Group A beta-hemolytic Streptococci accounting for 81% of PANDA cases) that leads to an abrupt onset of psychiatric manifestations [2, 5, 6].

**Figure 1:**
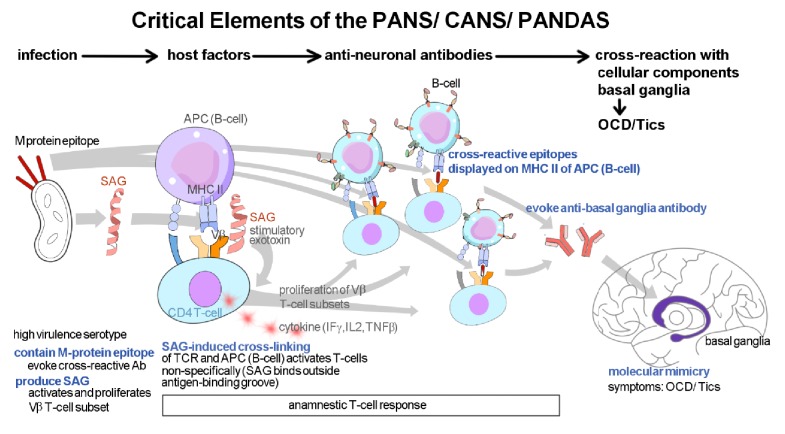
Hypothetical mechanism of postinfectious autoimmune neuropsychiatric syndromes. (1) Causative organism must contain M-protein epitopes that are capable of evoking antibody production that react with human neurons in the basal ganglia. They must also produce super-antigens (SAGs) that activate T-cell releasing cytokines (Interferon *γ*, Interleukin 2, and Tumor Necrosis Factor *β*) and proliferate T-cell (V subsets) resulting in anamnestic cell response. (2) T-cells bind to cross-reactive M-protein epitopes when displayed on surface MHC Class II molecules of B-cells. (3) B-cells are activated and (4) produce antibodies to M-proteins that cross-react with neuronal antigens in basal ganglia through the mechanism of molecular mimicry resulting in clinical manifestations such as OCD / tics [10].

Mechanistically, it is believed that microbes share antigenic determinants (sequence or structural similarities) with various human self-antigens referred to as “molecular mimicry" between host and microbe resulting in autoimmune reactions through these microbial cross-reactive autoantigens (Figure 1) [7–10]. For PANS/ CANS/ PANDAS, autoantibodies are believed to target dopamine receptors in susceptible hosts (D1-, D2-dopamine receptors) through the process of molecular mimicry causing anti-D2R/ D1R imbalance and greater sensitivity to central dopamine signaling leading to subsequent activation of calcium calmodulin-dependent kinase (CAMK2) which is manifested clinically by the abrupt onset of movement (e.g., tics) or neuropsychiatric symptoms (e.g., OCD with rituals or tics) [2, 11]. For PANDAs, these monoclonal antibodies demonstrate cross-reactivity with N-acetyl-beta-D-glucosamine (GlcNAc), the dominant epitope of the group A streptococcal (GAS) carbohydrate, and basal ganglia antigens [8, 11]. In further support of these autoantibodies and the autoimmune hypothesis of PANs are several reports that plasmapheresis, high dose pulse intravenous immunoglobulin replacement therapy, and corticosteroids result in immediate and strong suppression of acute postinfectious childhood OCD [3].

Although many viruses and bacteria have been linked to the initiation of an autoimmune response, identifying a particular virus or bacteria that is solely responsible for the induction of an autoimmune response is often complex because of the possibility for multiple infections being involved in priming the immune system and other infections triggering or exacerbating disease. Thus, no one viral or bacterial infection has been conclusively linked to the pathogenesis of CANS/ PANS [12]. Because a viral etiology besides bacterial has been linked to the pathogenesis of PANS, there is lack of evidence supporting the efficacy of antibiotic prophylaxis in patients fulfilling diagnostic criteria for PANS [13], whereas responses to immune-mediated treatments such as intravenous immunoglobulins or plasmapheresis have been supported by different studies [3, 14] but need reproducibility in larger trials.

## 4. Conclusion

Patient A was a challenging case because of her presentation with alarming psychosis and treatment failure with four antipsychotic medication regimens. This case illustrates the importance of establishing a broad differential diagnosis including underlying autoimmune encephalopathy, PANDAS/PANS, infection, and other immunologic disorders when evaluating patients presenting with OCD with rituals, severe anxiety, and psychotic symptoms. Unfortunately, neurology, rheumatology, and infectious disease consultants did not consider the diagnosis of PANs which delayed appropriate evaluation and treatment of our patient. This case emphasizes the importance of exploring nontraditional treatments for severe, treatment-resistant mental illness which requires a multidisciplinary approach [15]. Further research is warranted in larger populations to investigate pathomechanisms and treatment of PANS/PANDAS which will also serve to create greater awareness within the medical community about this disorder.

## Figures and Tables

**Table 1:** Results of patient A's anti-neuronal antibody panel.

	Anti-Dopamine Receptor D1 (titer)	Anti-Dopamine Receptor D2L (titer)	Anti-lysoganglioside GM1 (titer)	Anti-tubulin (titer)	CaM kinase II (% of baseline)
Patient results	1:8000	1:16,000	1:640	1:4,000	125
Normal Ranges	500-2,000	2,000-8,000	80-320	250-1,000	53-130
Normal Mean	1,056	6,000	147	609	95
Interpretation*∗*	Elevated	Elevated	Elevated	Elevated	Borderline

*∗*: elevation of any of the 5 assays is consistent with an autoimmune neurologic condition.
